# Entresto attenuates dexamethasone-induced metabolic dysfunction-associated steatotic features in rats: role of potential SGK1/FOXO3a regulatory pathway and cardio-metabolic axis

**DOI:** 10.3389/fphar.2026.1961803

**Published:** 2026-10-14

**Authors:** Mutni Kh. Alreshidi, Karim M. Saad, Marwa S. Zaghloul, Manar A. Nader

**Affiliations:** 1 Department of Pharmacology and Toxicology, Faculty of Pharmacy, Mansoura University, Mansoura, Egypt; 2 Faculty of Health Science Technology, Mansoura National University, Gamasa, Egypt

**Keywords:** DEXA-induced NASH, entresto, macrophage activation, mitochondrial biogenesis, SGK1, TRAF1

## Abstract

**Background:**

Prolonged or high dexamethasone (DEXA) exposure can promote steatohepatitis-like liver injury through insulin resistance, oxidative stress, inflammation, mitochondrial dysfunction, and fibrosis. Sacubitril/valsartan (Entresto) exhibits metabolic, anti-inflammatory, and antifibrotic properties that may protect against glucocorticoid-induced hepatic injury. The current study investigates the protective effects of Entresto against DEXA-induced hepatic injury in rats.

**Methods:**

Male rats were assigned into control, DEXA, and DEXA-treated groups receiving Entresto (30 or 60 mg/kg). Liver function, metabolic parameters, oxidative stress biomarkers, inflammatory and angiogenic mediators, fibrosis-related markers, and key signaling pathways were assessed using biochemical, molecular, histopathological, and immunohistochemical analyses.

**Results:**

DEXA administration induced hepatic injury, as evidenced by significant elevation of serum ALT, AST, and LDH levels and significant reduction in albumin concentration, accompanied by marked histopathological abnormalities. DEXA also promoted insulin resistance as reflected by increased fasting glucose, insulin, HOMA-IR and impaired OGTT. These changes were accompanied by metabolic dysregulation, including increased PEPCK and GSK expression, reduced glycogen synthase expression, elevated free fatty acids and oxidized LDL, and reduced PPAR-γ and PGC1-α. DEXA further altered IRS-1/AKT/JNK/p-AMPK/FOXO3 signaling, increased oxidative stress and mitochondrial dysfunction, enhanced inflammatory cytokine production and macrophage activation, and upregulated fibrosis-related mediators and renin-angiotensin-aldosterone system (RAAS) activation. In addition, DEXA increased SGK1, TRAF1, and SETD7 expression. Entresto treatment significantly attenuated DEXA-induced hepatic injury improved metabolic homeostasis and insulin sensitivity, enhanced antioxidant defenses, reduced inflammatory and fibrotic responses, and suppressed RAAS overactivation. These effects were associated with modulation of SGK1/FOXO3a signaling and activation of AMPK and PPAR γ-related pathways.

**Conclusion:**

ENT attenuates DEXA-induced metabolic liver injury with steatotic features by restoring insulin sensitivity, normalizing hepatic triglyceride accumulation, reducing oxidative stress, and modulating inflammatory and pro-fibrotic tissue markers, potentially through regulation of the SGK1/FOXO3a and cGMP/PKG/AMPK pathways crosstalk. These findings suggest that Entresto represents a promising therapeutic approach for mitigating glucocorticoid-induced steatohepatitis-like metabolic liver injury.

## 1 Introduction

Dexamethasone (DEXA), a synthetic glucocorticoid, is widely used in the management of numerous inflammatory and immune-mediated disorders, particularly chronic inflammatory conditions (Tiwari et al., 2024). However, prolonged exposure to high doses of DEXA is associated with multiple adverse effects, such as metabolic, hepatic, vascular, and homeostatic abnormalities (Gao, 2024; Polyzos and Targher, 2024; van Gerwen et al., 2023; Yao et al., 2024). These complications commonly manifest as insulin resistance (IR), glucose intolerance, dyslipidemia, oxidative stress, inflammation, and structural tissue alterations (Polyzos and Targher, 2024; van Gerwen et al., 2023). Collectively, these effects may contribute to the development of nonalcoholic fatty liver disease (NAFLD), a chronic liver disorder strongly associated with metabolic syndrome, type 2 diabetes mellitus (T2DM), dyslipidemia, and hypertension (Gaggini et al., 2013). NAFLD is characterized by hepatic steatosis, and may progress to nonalcoholic steatohepatitis (NASH), fibrosis, cirrhosis, and hepatocellular carcinoma (Lonardo et al., 2013). Although NASH is continuously emerging, recent progress in pharmacological options for NASH remains limited. Hence, there is a need for additional therapeutic strategies to target metabolic, inflammatory, and fibrotic pathways in NASH.

Mechanistically, glucocorticoid-induced IR is associated with suppression of insulin signaling and activation of gluconeogenic enzymes. These alterations promote hepatic glucose overproduction, increased hepatic lipogenesis, impaired fatty acid oxidation, and broader metabolic imbalance. At the cellular level, mitochondrial dysfunction represents a key contributor to disease progression and is accompanied by excessive reactive oxygen species (ROS) generation. Disrupted mitochondrial biogenesis and impaired energy metabolism further contribute to hepatocyte injury and progression of steatohepatitis-like pathology (Begriche et al., 2006; Friedman and Nunnari, 2014). Inflammatory and immune mechanisms also contribute to NASH progression in this model. DEXA-induced metabolic injury may involve dysregulated macrophage activation and altered inflammatory signaling, in addition, CD4^+^ T cells and immunoglobulin (Ig)M alterations reflect systemic inflammatory activation associated with metabolic stress (Martinez and Gordon, 2014; Sica and Mantovani, 2012; Tacke and Zimmermann, 2014).

Serum and glucocorticoid-regulated kinase 1 (SGK1), a downstream glucocorticoid-responsive kinase, contributes to metabolic dysregulation by modulating glucose transport, sodium handling, and inflammatory responses. SGK1 activation has also been implicated in IR and fibrotic signaling, thereby glucocorticoid-driven metabolic stress to tissue remodeling and hepatic injury (Lang and Shumilina, 2013; Manning and Toker, 2017).

Entresto (ENT), a first-in-class angiotensin receptor–neprilysin inhibitor (ARNI) comprised of sacubitril and valsartan, is widely used in the management of heart failure. Beyond its cardiovascular effects, accumulating evidence suggests that ENT exerts pleiotropic actions, including improving of insulin sensitivity, attenuation of oxidative stress, suppression of inflammatory responses, modulation of fibrogenic pathways, and restoration of metabolic homeostasis (Aroor et al., 2021; Cloro et al., 2022; Seferovic et at., 2020). However, its potential protective effects against DEXA-induced NASH remain poorly understood.

Therefore, the current study aims to investigate the protective effects of ENT against DEXA-induced NASH in rats, with particular focus on hepatic dysfunction, insulin resistance, oxidative stress, macrophage activation, inflammatory responses, fibrotic remodelling, and related signaling pathways.

## 2 Materials and methods

### 2.1 Drugs and chemicals

DEXA sodium phosphate was obtained as a commercially available preparation manufactured by Amriya Pharmaceutical Industries (Alexandria, Egypt) in ampoules containing 8 mg/2 mL (USP grade). Before administration, DEXA was diluted in sterile 0.9% normal saline. ENT was gifted by Novartis (Switzerland) and suspended 0.5% (w/v) carboxymethylcellulose (CMC) solution for oral administration *via* gavage. All other chemicals and reagents used in the study were of analytical grade.

### 2.2 Experimental animals

Young adult male Wistar rats (7–8 weeks old, 180 ± 20 g) were used in this study. The animals were obtained from the Egyptian Organization for Biological Products and Vaccines (VACSERA, Giza, Egypt). Upon arrival, rats were allowed to acclimatize for 1 week under controlled laboratory conditions (temperature: 25 °C ± 2 °C; relative humidity: 50%–60%; 12-h light/dark cycle). Animals were housed in standard cages (six rats per cage) with free access to standard chow and water. All experimental procedures were approved by the Mansoura University Animal Care and Use Committee (MU-ACUC; Protocol No: PHARM. MS.25.01.131) and conducted in accordance with the ARRIVE guidelines.

### 2.3 Experimental design

Animals were randomly assigned to four experimental groups using a computer-generated randomization scheme. Investigators responsible for outcome assessment and data analysis were blinded to group allocation. A total of 24 male Wistar rats were divided as follows ( n=6 per group): Control, DEX, ENT 30 + DEX, and ENT 60 + DEX. No animal mortality or exclusions were recorded throughout the study period. All animals ( n=6 per group) were evaluated for general phenotypic parameters and baseline serum biomarkers. For specific downstream molecular mechanisms, quantitative immunohistochemistry, and pathway-specific protein assays, a representative subset of n=4 per group was randomly selected and analyzed. The sample size is 3:5 and was determined using the Resource Equation approach, ensuring adequate statistical power ( power=0.80,α=0.05 ) (Arifin and Zahiruddin, 2017; Charan and Kantharia, 2013). A total of 24 rats were divided as follows:

- Control group: rats received vehicles only for 13 consecutive days.
- DEX group: rats received 0.5% CMC for 7 days, followed by intraperitoneal (i.p.) injection of DEX (8 mg/kg/day) for the subsequent 6 days (Alharbi et at., 2026).
- ENT 30 + DEX group: rats received ENT (30 mg/kg/day, orally) for 13 days, DEX (8 mg/kg/day, i. p.) was administered 60 min after ENT, starting on day 8 for 6 days.
- ENT 60 + DEX group: rats received ENT (60 mg/kg/day, orally) for 13 days; DEX was administered as described above.

The selected doses of Entresto (30 and 60 mg/kg/day, Orally) were chosen according to previously published experimental studies demonstrating the pharmacological efficacy and safety of sacubitril/valsartan in rat models of inflammatory, renal, metabolic, and cardiovascular disorders (Abdel-Latif et al., 2020; El-Sayed and El-Sawy, 2022; Kusaka et at., 2018; Mustafa et al., 2022).

Body weight was monitored throughout the experimental period. The percentage change in body weight was calculated relative to baseline values recorded on day 1. At the end of the experiment, blood samples were collected from the retro-orbital venous plexus under deep anesthesia (secobarbital, 50 mg/kg, i. p.). Serum was separated by centrifugation (3,000 rpm, 15 min, 4 °C) and stored at −20 °C. Animals were then euthanized by cervical dislocation under deep anesthesia, and liver tissues were excised, rinsed with ice-cold saline, dried, weighed and processed for subsequent biochemical and histological analyses.

### 2.4 Oral glucose tolerance test (OGTT)

An oral glucose tolerance test was performed on the final day of the experiment. Following an overnight fast (10 h), rats were administered glucose solution (40%, 2 g/kg body weight) orally. Blood glucose levels were measured at 0 (fasting), 30, 60, 120, and 180 min post-glucose administration using a validated glucometer (Accu-Chek®, Roche Diagnostics, Basel, Switzerland).

### 2.5 Assessment of fasting insulin and glucose

After overnight fasting, blood glucose was measured *via* tail vein sampling. Serum insulin levels were determined using a commercial electrochemiluminescence assay kit (Roche Elecsys, Mannheim, Germany).

### 2.6 Determination of insulin resistance (IR)

IR was assessed using the homeostatic model assessment of IR (HOMA IR), which is calculated according to the following equation as previous (Abdelmageed et at., 2019):

$$HOMA-IR=\frac{Fasting\;Serum\;Insulin\;\left(mu/L\right)\times Fasting\;Serum\;Glucose\;\left(mmol/L\right)}{22.5}$$

### 2.7 Liver function tests

Serum levels of alanine aminotransferase (ALT), aspartate aminotransferase (AST), lactate dehydrogenase (LDH) and albumin (Alb) were measured using commercial diagnostic kits (AGAPPE Diagnostics, India) to evaluate hepatic injury.

### 2.8 Oxidative stress markers

Hepatic levels of MDA were determined as an index of lipid peroxidation, while SOD were assessed to evaluate antioxidant status using commercially available kits (Biodiagnostic, Egypt). Additionally, hepatic levels of 8-hydroxydeoxyguanosine (8-OHdG) were assessed using commercially available kits (Cat. No. E-EL-0028, Elabscience, United States).

### 2.9 Determination of total protein content

Total protein content in liver homogenates was quantified using the Bradford method with a Genei protein assay kit (Bangalore, India).

### 2.10 Determination of hepatic ATP level

Hepatic ATP was measured using a colorimetric assay kit obtained from Elabscience, United States, Cat. No. E-BC-K157-S.

### 2.11 ELISA analysis

Observing the manufacturer’s guidelines, the following proteins were identified using commercially available ELISA kits.

**Table:** 

Marker	Catalog no.	Company (Country)
Hepatic markers		
8-hydroxydeoxyguanosine (8-OHdG)	E-EL-0028	Elabscience (United States)
Interferon-γ (IFN-γ)	E-EL-R0009	
Immunoglobulins M (IgM)	E-EL-R3054	
Oxidized low-density lipoprotein (Ox-LDL)	E-EL-R3034	
Heme oxygenase-1 (HO-1)	E-EL-R0488	
Protein kinase B (AKT)	MBS3807575	MyBioSource (United States)
Cluster of differentiation (CD4)	MBS721241	
CD34	MBS3807594	
Insulin receptor substrate-1 (IRS-1)	MBS2706135	
Nuclear factor kappa-light-chain-enhancer of activated B cells (NF-κB p65)	MBS7226224	
Fatty acid-binding protein (FABP)	MBS264324	
Rat epidermal growth factor receptor (EGFR)	MBS269875	
Transforming growth factor-β1 (TGF-β1)	MBS824788	
Nuclear factor erythroid 2-related factor 2 (Nrf2)	MBS752046	
Phosphoinositide 3-kinase (PI3K)	MBS9356979	
Peroxisome proliferator-activated receptor gamma (PPAR-γ)	MBS2701257	
CD86	MBS2503027	
CD163	MBS2024373	
Free fatty acids (FFA)	MBS733451	
Glycogen synthase kinase-3 alpha (GSK-3α)	MBS2513979	
CD80	LS-F56244	LS bio (United States)
Musculoaponeurotic fibrosarcoma oncogene homolog (c-Maf)	LS-F16315	
Aldosterone	ELK9729	ELK biotechnology (United States)
Peroxisome proliferator-activated receptor-gamma coactivator-1 alpha (PGC1α)	ELK6194	
Dyamin-related protein 1 (Drp-1)	ELK0215	
Rat triglycerides (TAGs) ELISA kit	ELK8384	
CD206	NBP3-08152	Novus biologicals (United States)
Glycogen synthase (GS)	RE2350R	Reed biotech (China)
Glucose-6-phosphatase (G6Pase)	ER0967	Fine test (China)
Phosphoenolpyruvate carboxykinase (PEPCK)	RAT09161	Assay genie (Ireland)
c-Jun N-terminal kinase (JNK)	RTFI00924	
Forkhead box protein O1 (FOXO1)	RTDL00393	
Vascular endothelial growth factor (VEGF)	ab100786	Abcam (United Kingdom)
AMP-activated protein kinase alpha 1 (AMPKα1)	DYC3197-2	R&D systems (United States)
Plasminogen activator inhibitor-1 (PAI-1)	CSB-E07948r	Cusabio (United States)
Fibronectin	CSB-E04553r	
Serum markers		
Angiotensin 2	NBP3-50013	R&D systems (United States)
Renin	MBS041519	MyBioSource (United States)
Brain natriuretic peptide (BNP)	E-EL-R0126	Elabscience (United States)
Atrial natriuretic peptide (ANP)	NBP2-68221	Novus biologicals (United States)

### 2.12 Histopathological examination

Formalin-fixed liver tissues were embedded in paraffin, sectioned (5 µm), and stained with hematoxylin and eosin (H&E). Histological assessment was performed in a blinded manner using a light microscope equipped with a digital imaging system. Steatosis and inflammatory activity were scored using established semiquantitative criteria based on the Kleiner scoring system (Kleiner et al., 2005).

### 2.13 Immunohistochemistry (IHC)

Liver sections were processed for immunohistochemical detection of FOXO3, TRAF1 and SETD7 (MyBioSource, California, United States, Cat. No. MBS2523042, MBS2535351, and MBS2526288 respectively) and SGK-1 (ELK Biotechnology CO. Ltd., TX, United States, Cat. No. EM1264)**.** After antigen retrieval and blocking, sections were incubated with primary antibodies overnight at 4 °C, followed by HRP-conjugated secondary antibodies. DAB was used for visualization. Quantitative analysis was performed using ImageJ software, and results were expressed as percentage area of positive staining.

### 2.14 Statistical analysis

Sample size was determined based on power analysis (power = 0.8, α = 0.05). The sample size for phenotypic baseline and standard serum biochemical profiles was 6 animals per group (n = 6 biological replicates). For specific advanced downstream protein expression assays, quantitative immunohistochemistry, and molecular pathway evaluations, data were obtained from a representative subset of 4 animals per group (n = 4 biological replicates), selected in compliance with the Resource Equation method for animal research optimization. Data were tested for normality using the Shapiro–Wilk test. Parametric data were expressed as mean ± SD and analyzed using one-way ANOVA followed by Tukey’s *post hoc* test. Biological replicates ( n ) were strictly distinguished from technical replicates; technical replicates (e.g., duplicate assay wells for ELISA or multiple microscopic fields per section) were averaged to yield a single value per animal prior to statistical testing. Statistical analysis was conducted using GraphPad Prism (version 8.0), with significance set at p < 0.05.

## 3 Results

Based on the initial biochemical and histopathological assessments, both tested doses of ENT demonstrated significant protective effects against the induced tissue injury. However, the higher dose consistently exhibited superior efficacy, as evidenced by more pronounced improvements in functional biomarkers and histological architecture. Therefore, to optimize resource utilization and facilitate a more focused mechanistic investigation, some molecular analyses were performed using the higher ENT dose only. This approach was adopted to elucidate the molecular pathways underlying the maximal therapeutic response observed in the dose-screening phase and to identify the key signaling mechanisms responsible for its protective effects.

### 3.1 Effect of ENT (30, 60 mg/kg, orally) on serum hepatic injury markers, liver weight indices and histopathological architecture in DEXA-induced hepatic injury

Serum ALT, AST, and LDH activities were significantly increased in the DEXA group compared to control rats. For the 30 or 60 mg/kg dose, ENT significantly reduced the levels of liver enzymes compared to the DEXA group (Figures 1A–C). DEXA significantly increased liver weight, and the relative liver/body weight index compared to control rats. Both doses of ENT failed to significantly decrease the DEXA-induced increase in liver and liver/body weight index (Figures 1D,E).

**FIGURE 1:**
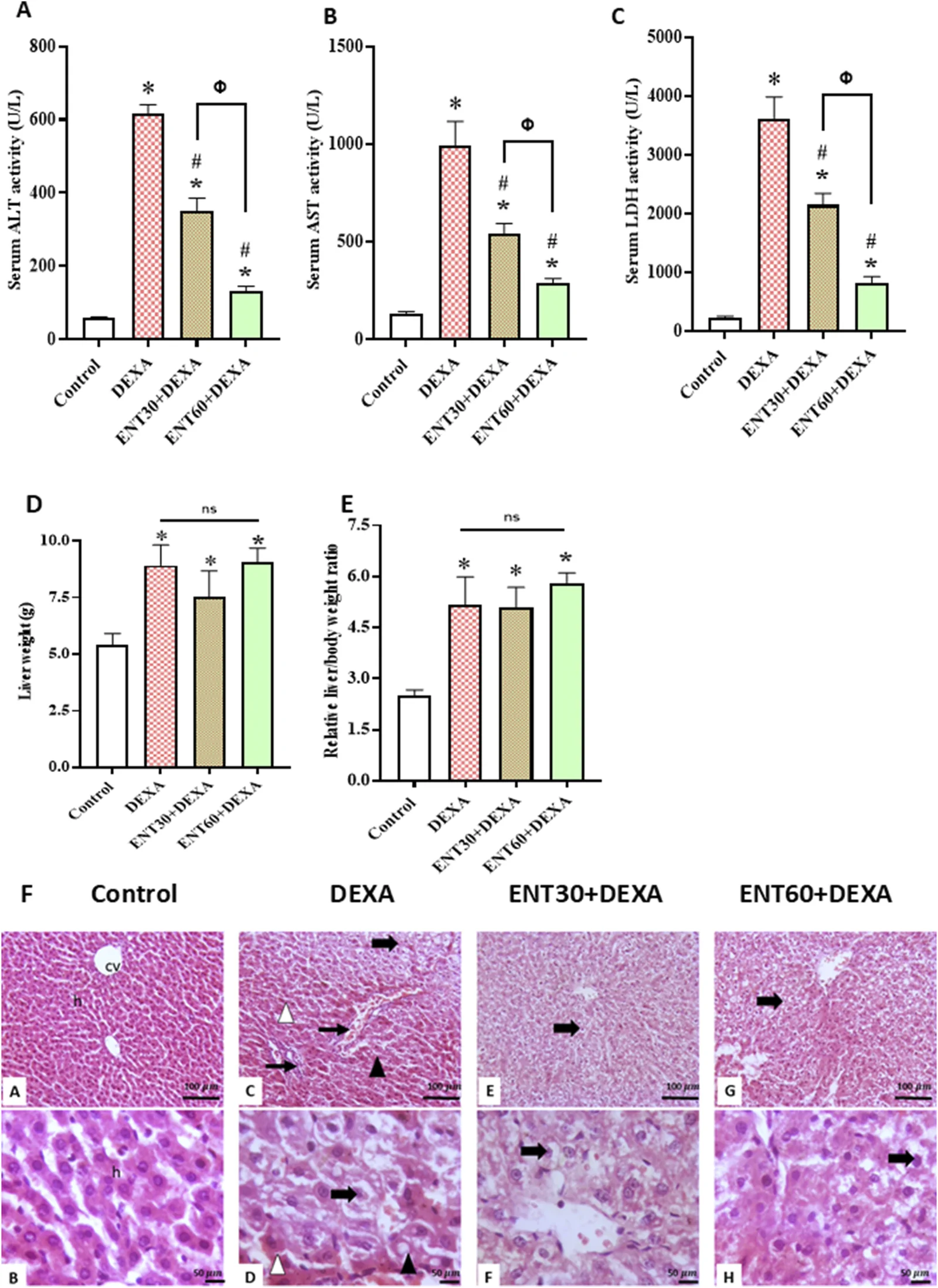
Effect of ENT (30, 60 mg/kg, Orally) on serum hepatic injury markers, liver weight indices, and histopathological architecture in DEXA-induced hepatic injury. **(A–C)** Serum ALT, AST, and LDH activities were measured as biochemical markers of hepatocellular injury. **(D,E)** Liver weight and liver/body weight index were assessed to evaluate gross hepatic architecture and histopathological alterations among the experimental groups. **(F)** H&E-stained liver sections photomicrographs. Cv: central vein, H: hepatocytes, Black thick arrows: hepatocellular swelling, thin arrows: fibrosis with inflammation, black arrowheads: intracytoplasmic vacuoles (steatosis), arrowheads: hepatocellular necrosis. Image magnification = 100x: Bar 100 μm , 400x: Bar 50 μm . Data are expressed as mean ± SD (n = 4). ENT: Entresto; DEXA: Dexamethasone; Alb: Albumin; ALT: Alanine aminotransferase; AST: Aspartate aminotransferase; DEXA: Dexamethasone; LDH: Lactate dehydrogenase; H&E: hematoxylin-eosin.

H&E stained-liver sections revealed microscopic pictures comparing the effects of low-dose vs. high-dose ENT treatment in DEXA toxified rats. The control group exhibited normal liver architecture, with a central vein, portal tracts, and hepatic plates. The portal tract showed portal venule, hepatic arteriole, and bile ductile, surrounded by hepatocytes and blood sinusoids. The DEXA group liver tissues showed diffuse hepatocellular necrosis characterized by severe hypereosinophic cytoplasm with pyknotic nucleus (necrosis) admixed with moderate hepatocellular swelling and scattered numerous intracytoplasmic vacuoles together with multifocal mild perivascular fibrosis with low numbers of inflammatory cells. ENT30 + DEXA revealed moderate hepatocellular swelling and vacuolation without inflammatory infiltration. ENT60 + DEXA showed multifocal mild to moderate hepatocellular degeneration and vacuolation without any inflammatory cells (Figure 1F).

### 3.2 Effect of ENT (30, 60 mg/kg, orally) on DEXA-induced alterations in glucose homeostasis and hepatic insulin/metabolic signaling

DEXA administration induced a significant disruption in glucose homeostasis as evident by significant increase of AUC of OGTT and FBG compared to control. Higher dose of ENT only significantly reduced both markers compared to DEXA group, however, lower ENT dose only significantly reduced FBG, with a non-significant decrease in AUC of OGTT compared to DEXA group (Figures 2A–C).

**FIGURE 2:**
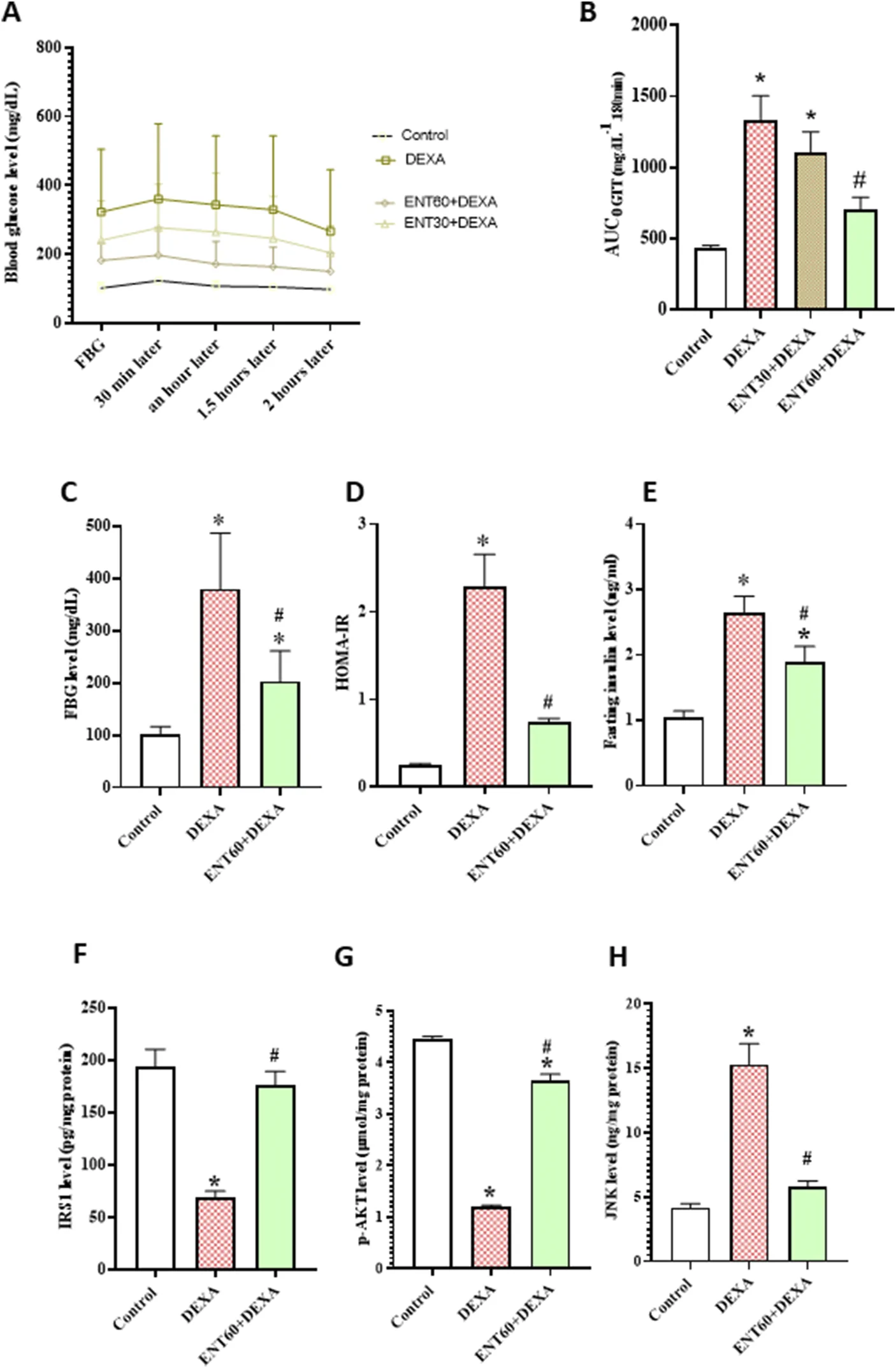
Effect of ENT (30, 60 mg/kg, Orally) on DEXA-induced alterations in glucose homeostasis and hepatic insulin/metabolic signaling. **(A)** OGTT curve and **(B)** OGTT_AUC_ were used to assess glucose tolerance. **(C–E)** Fasting blood glucose, HOMA-IR and serum insulin were measured as systemic metabolic indices. **(F–H)** Hepatic IRS-1, p-AKT, and total JNK levels were assessed to evaluate insulin-associated and stress-related signaling. Data are expressed as mean ± SD (n = 4). ENT: Entresto; DEXA: Dexamethasone; AUC: Area under the Curve; DEXA: Dexamethasone; OGTT: oral glucose tolerance test: HOMA-IR: homeostatic model assessment of insulin resistance.

Since the higher ENT dose was more effective, and to limit resource utilization, we measured insulin levels, HOMA-IR, hepatic IRS-1, p-AKT, and p-JNK only for ENT (60 mg/kg, Orally), but not for the lower dose. DEXA-induced a significant decrease in hepatic levels of IRS1/p-AKT and increased JNK level compared to the control group. Treatment with ENT (60 mg/kg, Orally) significantly restored hepatic levels of IRS1/p-AKT/JNK compared to the DEXA group (Figures 2D–H).

### 3.3 Effect of ENT (60 mg/kg, orally) on DEXA-induced alterations in hepatic lipid metabolism and lipotoxicity

Rats administered DEXA showed significantly higher levels of hepatic FFA, Ox-LDL, and FABP, TAGs as matched to the control group. ENT (60 mg/kg, Orally) significantly decreased hepatic levels of those biomarkers compared to the DEXA group (Figures 3A-D).

**FIGURE 3:**
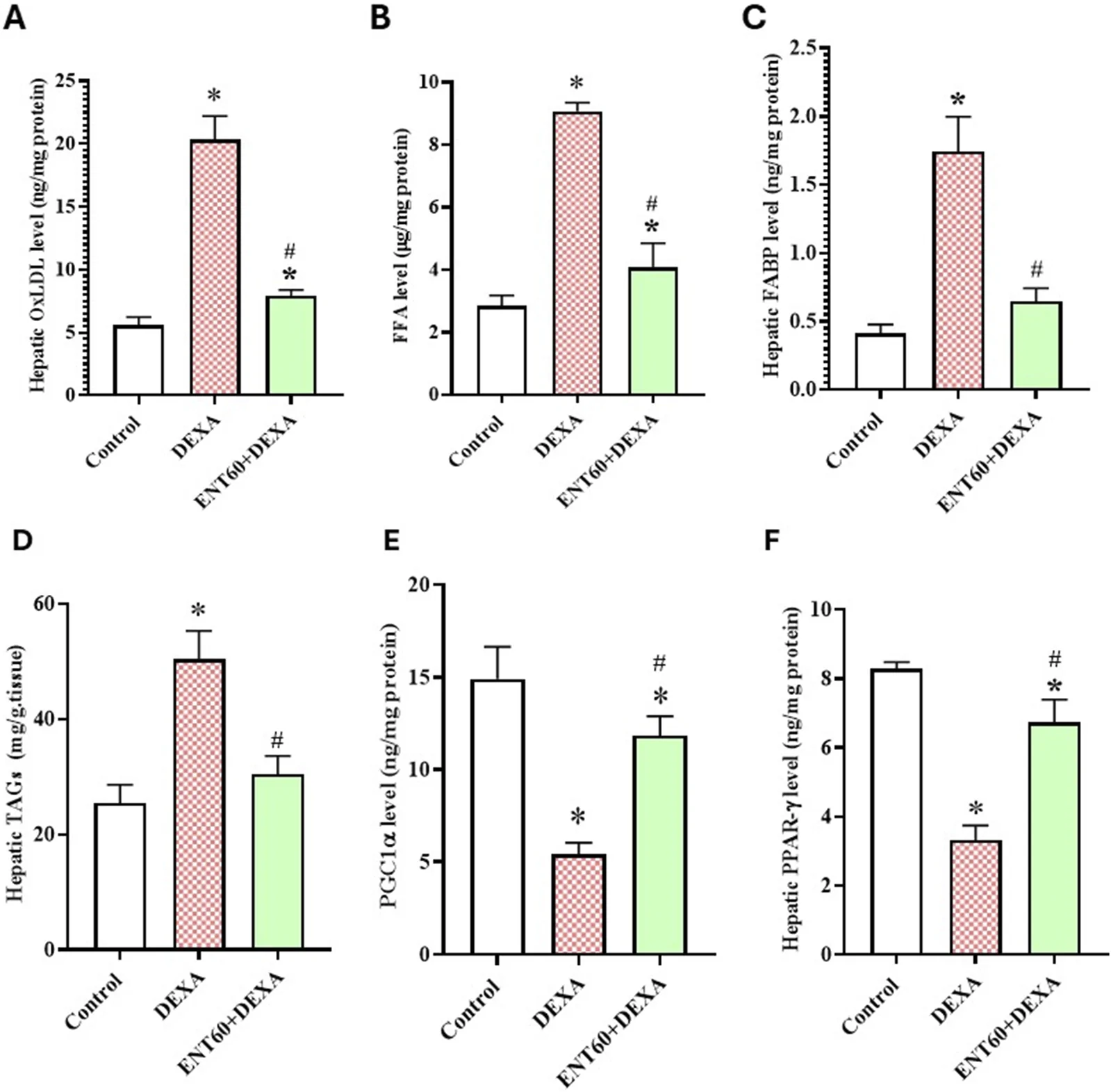
Effect of ENT (60 mg/kg, orally) on DEXA-induced alterations in hepatic lipid metabolism and lipotoxicity. **(A–D)** Hepatic OxLDL, FFA, and FABP, TAGs levels were measured as markers of oxidized lipid accumulation, fatty-acid burden, and fatty-acid handling. **(E,F)** Hepatic PGC-1α and PPAR-γ levels were assessed as metabolic regulatory markers related to lipid metabolism and hepatic metabolic adaptation. Data are expressed as mean ± SD (n = 4). ENT: Entresto; DEXA: Dexamethasone; FFA: Free fatty acids; PGC1α: Peroxisome proliferator-activated receptor-gamma coactivator-1 alpha: Ox-LDL: Oxidized low-density lipoprotein; PPAR-γ: Peroxisome proliferator-activated receptor gamma; FABP: Fatty acid-binding protein; TAGs: triglycerides.

Regarding PGC1α and PPAR-γ levels, as demonstrated in Figures 3D-F, the DEXA group showed a significant decrease in levels of PGC1α, and PPAR-γ levels compared to the control group. While the ENT60 + DEXA group showed a significant increase in expression of both biomarker levels when compared to the DEXA group.

### 3.4 Effect of ENT (60 mg/kg, orally) on DEXA-induced alterations in hepatic oxidative stress, mitochondrial dynamics, and energy sensing

DEXA induced a significant elevation in hepatic MDA and 8-OHdG levels, as indicators to oxidative stress and oxidative DNA damage, respectively, and a significant reduction in SOD, Nrf2, and HO1 levels, as indicators to antioxidant capacity, compared to control group. On the other hand, the pre-treatment with ENT60 showed a significant reduction in oxidative stress markers and elevation in antioxidant biomarkers compared to DEXA group (Figures 4A–E).

**FIGURE 4:**
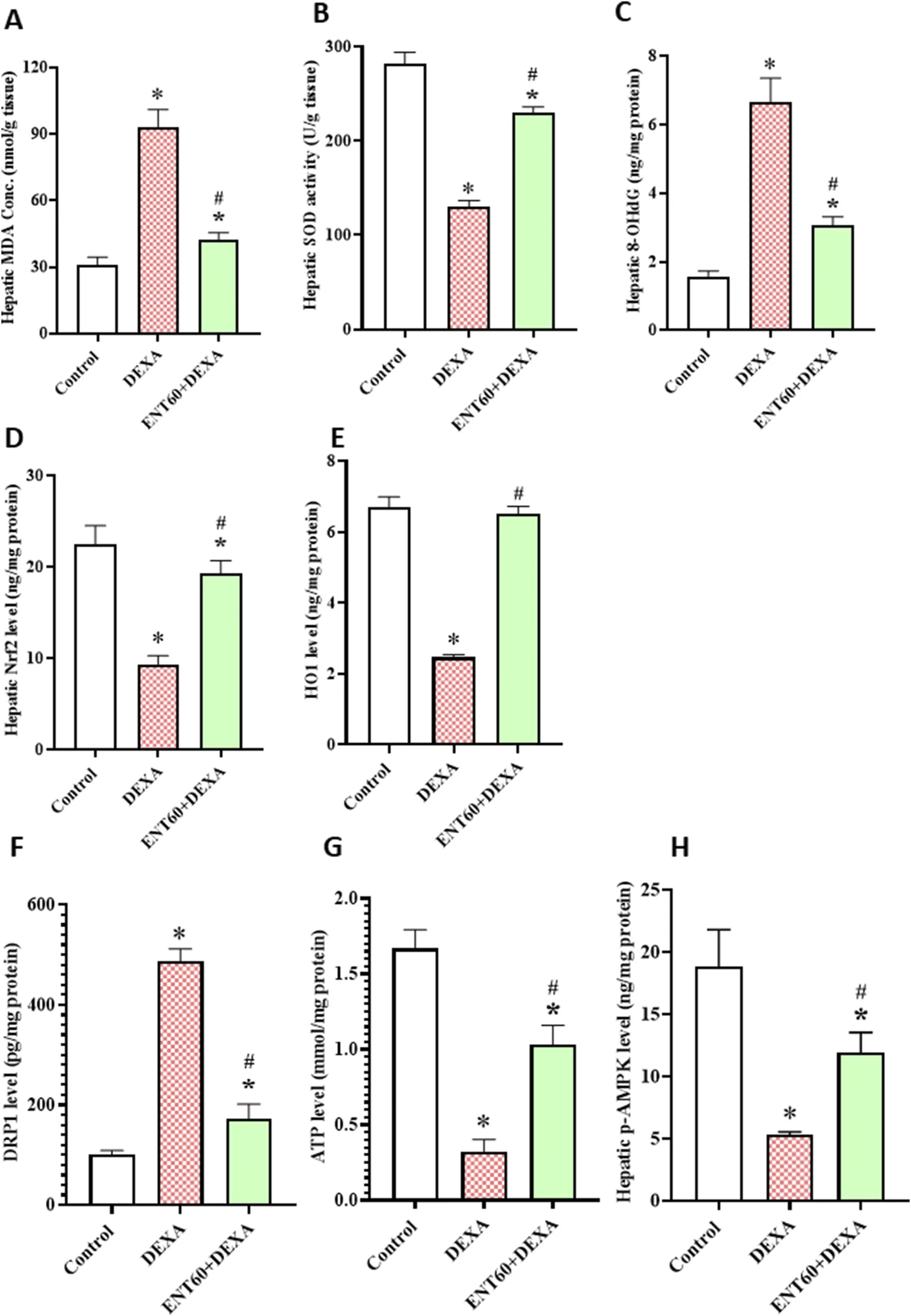
Effect of ENT (60 mg/kg, orally) on DEXA-induced alterations in hepatic oxidative stress, mitochondrial dynamics, and energy sensing. **(A–C)** Hepatic MDA, SOD, and 8-OHdG levels were measured to evaluate lipid peroxidation, antioxidant capacity, and oxidative DNA damage. **(D,E)** NRF2 and HO-1 were assessed as antioxidant defense markers. **(F–H)** DRP1, ATP, and p-AMPK were measured to evaluate mitochondrial dynamics, bioenergetic status, and energy-sensing signaling. Data are expressed as mean ± SD (n = 4). ENT: Entresto; DEXA: Dexamethasone; 8-OHdG: 8-hydroxydeoxyguanosine; SOD: Superoxide Dismutase; MDA: Malondialdehyde; Nrf2: Nuclear factor erythroid 2-related factor 2; HO1: Heme oxygenase1; DRP1: Dynamin-related protein 1; ATP: Adenosine triphosphate; p-AMPK: phosphorylated AMP-activated protein kinase.

For mitochondrial dynamics and energy sensing, DEXA resulted in a significant elevation in hepatic DRP1 levels and a significant reduction in ATP levels and p-AMPK in compared to control groups. On the other hand, the pre-treatment with ENT60 caused a significant decrease in DRP1, and significant elevation of ATP and p-AMPK levels compared to DEXA group (Figures 4F–H).

### 3.5 Effect of ENT on DEXA-induced alterations in hepatic TNF/NF-κB/TRAF1 signaling, macrophage polarization, and adaptive immune activation

TNF- α and NF-κB were assessed as markers of hepatic inflammatory signaling, while TRAF1 immunohistochemistry was used to localize and quantify tissue inflammatory activation. CD80 and CD86 were evaluated as markers of M1 activation, whereas c-MAF, CD163 and CD206 were assessed as M2 macrophage markers. CD4, IFN-γ, and IgM were included to evaluate adaptive and humoral immune involvement.

DEXA induced a significant increase in M1-like/macrophage activation, as evident by a significant increase in hepatic CD80 and C86 compared to the control group. Moreover, DEXA induced a significant increase in inflammatory signaling, as evident by a significant increase in hepatic TNF-α, and NF-κB p65 compared to control group. Treatment with ENT (60 mg/kg, Orally) significantly modulated the intrahepatic microenvironment, as evidenced by a statistically significant shift in the ratio of M2/M1-like surface markers (CD163/CD80) and reduced levels of TNF-α, and NF-κB p65 compared to the control group (Figures 5A–D).

**FIGURE 5:**
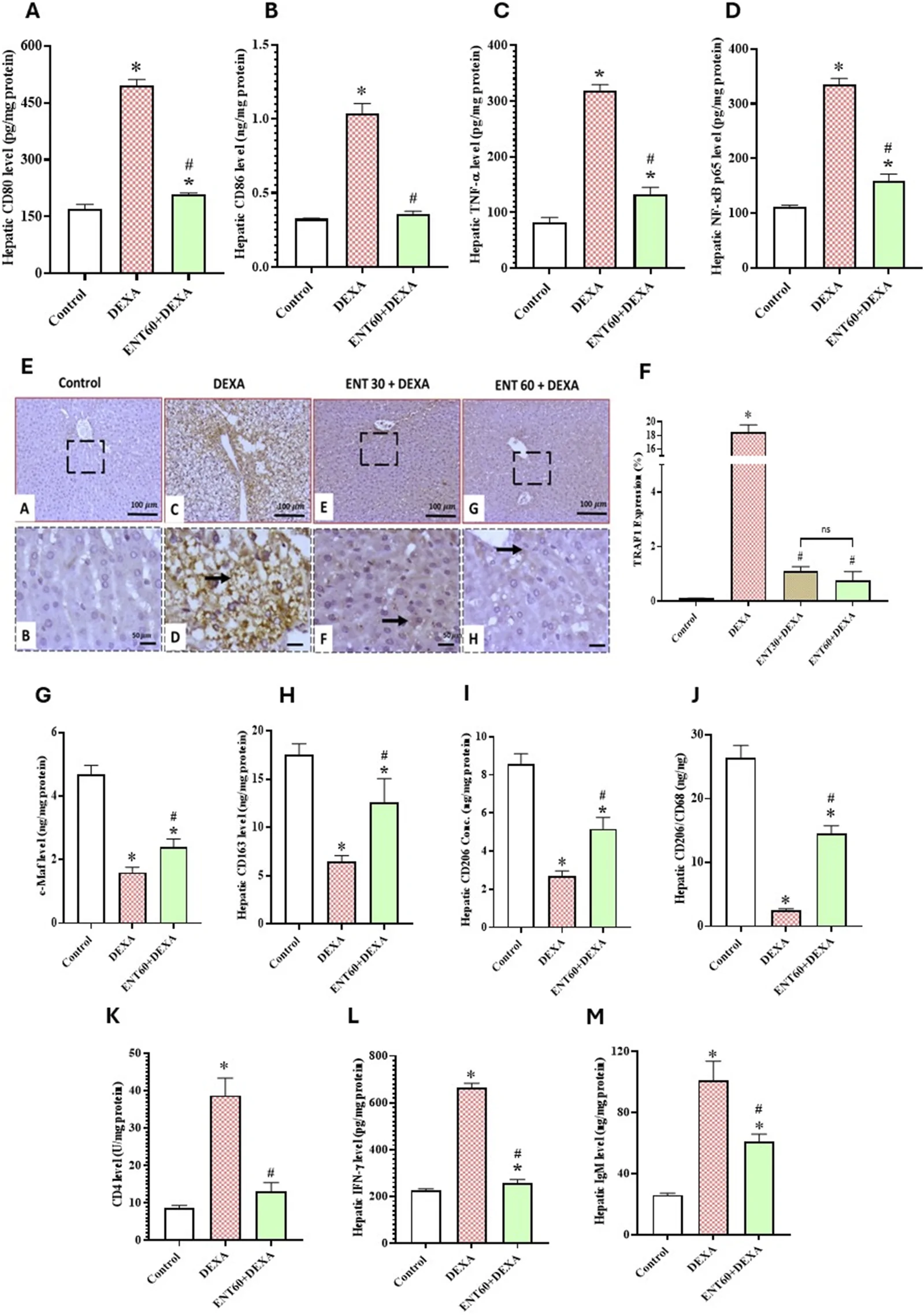
Effect of ENT on DEXA-induced alterations in hepatic TNF/NF-κB/TRAF1 signaling, macrophage polarization, and adaptive immune activation. **(A,B)** CD80 and CD86 were assessed as markers of M1-like macrophages. **(C–F)** TNF-α, NF-κB, and TRAF1 immunohistochemistry were used to evaluate hepatic inflammatory signaling. **(G–I)**, **(J)** The CD206/CD86 ratio was quantified as an indicator of the balance between anti-inflammatory (M2) and pro-inflammatory (M1) macrophage polarization within the hepatic microenvironment. CMAF, CD163, and CD206 were measured as M2-like macrophage-associated/reparative markers. **(J–M)**, CD4, IFN-γ, and hepatic IgM were assessed to evaluate adaptive and humoral immune activation. Data are expressed as mean ± SD (n = 4). ENT: Entresto; DEXA: Dexamethasone; CD: Cluster of differentiation; TNF: Tumor necrosis factor; NF-κB: Nuclear factor-κB; IgM: Immunoglobulin M; c-Maf: Musculoaponeurotic Fibrosarcoma Oncogene Homolog. TRAF1: TNF receptor-associated factor 1; IFN-γ = Interferon-gamma.

Representative IHC expression and tissue localization of TRAF1 in hepatic sections of normal group showed negative immunostaining of TRAF1 in hepatocytes. DEXA group showed moderate strong cytoplasmic expression in vacuolated and steatotic perivascular hepatocytes. ENT30 group showed occasional faint cytoplasmic immunostaining in hepatocytes and Kupffer cells. ENT60 group showed few, occasionally faint cytoplasmic immunopositive expressions in hepatocytes. Additionally, DEXA administration significantly induced the hepatic expression of TRAF1 compared to control group, the administration of ENT at both doses profoundly declined the protein expression and tissue localization levels of TRAF1, competed to the DEXA group (Figures 5E,F).

For M2 polarization markers, DEXA induced a significant decrease in hepatic c-MAF, CD163 and CD206 compared to control group. Treatment with ENT (60 mg/kg, Orally) significantly elevated M2-markers compared to DEXA group (Figures 5G–I). Furthermore, DEXA-treated rats showed a significant reduction in the CD206/CD86 ratio compared with controls. Co-treatment with Entresto (60 mg/kg, orally) significantly increased the CD206/CD86 ratio relative to DEXA alone, although it remained significantly lower than control levels (Figure 5J).

For adaptive/humoral immune involvement, DEXA induced a significant increase in hepatic CD4, as a marker of T-helper cells, IFN-γ, a pro-inflammatory cytokine that can also be released from Th1 cells, and hepatic IgM. ENT (60 mg/kg, Orally) treatment significantly attenuated DEXA-induced increase in hepatic CD4, IFN-γ and IgM compared to DEXA group (Figures 5K–M).

### 3.6 Effect of ENT (30 and 60 mg/kg, orally) on DEXA-induced alterations in hepatic SGK1/SETD7/FOXO3a signaling, gluconeogenesis, and glycogen metabolism

Representative IHC expression and localization of SGK1 (Figure 6A) in hepatic sections of normal group showed mild cytoplasmic immunoexpression of SGK1 in hepatocytes and Kupffer cells. Meanwhile, DEXA group showed marked upregulation of SGK1 with strong cytoplasmic with occasional nuclear immunoreactivity in steatotic hepatocytes and perivascular inflammatory cells. ENT30 showed occasional faint cytoplasmic immunostaining in hepatocytes with scattered positivity in Kupffer cells. ENT60 group showed scattered faint cytoplasmic immunopositive expression in hepatocytes and Kupffer cells. DEXA significantly induced the hepatic expression of SGK1 compared to control group. In contrast, coadministration of ENT in DEXA-treated rats was associated with decrease in accumulation of SGK1 compared to DEXA-treated rats. Interestingly, the effect of high dose of ENT resulted in a marked reduction compared to low dose, restoring it near control levels (Figure 6B).

**FIGURE 6:**
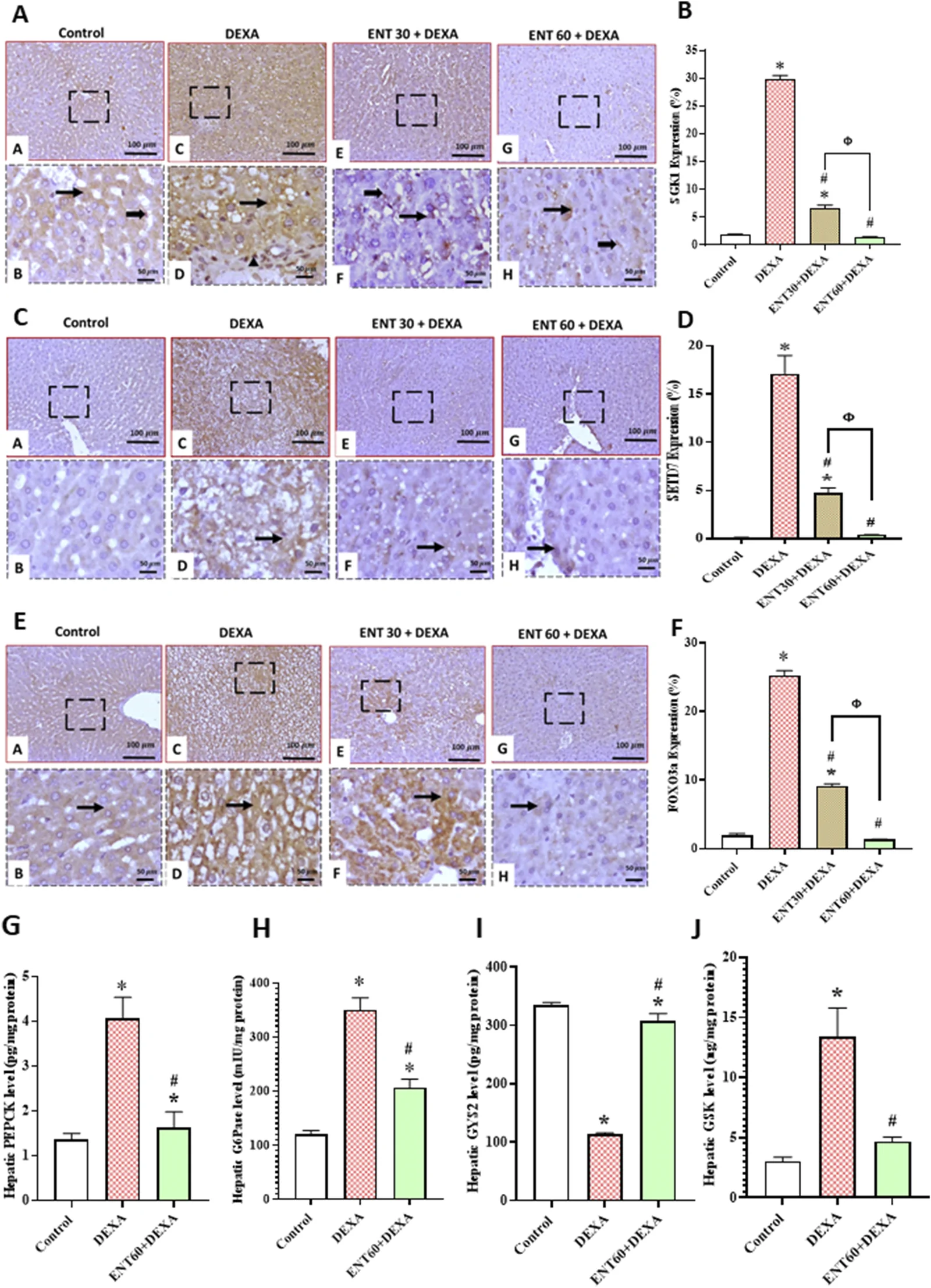
Effect of ENT (30 and 60 mg/kg, orally) on DEXA-induced alterations in hepatic SGK1/SETD7/FOXO3a signaling, gluconeogenesis, and glycogen metabolism. **(A–F)** SGK1, SETD7, and FOXO3a immunohistochemistry was performed to assess hepatic metabolic-stress and FOXO-related signaling. **(G,H)** PEPCK and G6Pase were measured as gluconeogenic enzymes. **(I,J)** GYS2 and total GSK were assessed as markers related to hepatic glycogen-metabolic regulation. Data are expressed as mean ± SD (n = 4). ENT: Entresto; DEXA: Dexamethasone; SGK1: glucocorticoid-regulated kinase 1; SETD7: SET domain-containing lysine methyltransferase 7; FOXO3a: Forkhead box O3a.; PEPCK: Phosphoenolpyruvate carboxykinase; G6Pase: Glucose-6-phosphatase; GYS2: Glycogen synthase 2; GSK: glycogen synthase kinase.

Additionally, representative IHC expression and localization of SETD7 (Figure 6C) in hepatic sections of the normal group showed the absence to few faint immunostainings of SETD7 in hepatocytes. The DEXA group showed moderate to strong cytoplasmic and perinuclear immunoreactivity in steatotic hepatocytes. ENT30 showed negative to occasional immunostaining in hepatocytes. ENT60 showed occasional faint cytoplasmic immunopositive expression in hepatocytes and Kupffer cells. DEXA significantly induced the hepatic expression of SETD7 compared to the control group; the administration of ENT at both doses effectively decreased the hepatic expression levels of SETD7, comparable to the DEXA group. Moreover, ENT60 treatment resulted in a significant increase in hepatic expression of SETD7 when compared to the ENT30-treated group (Figure 6D).

Representative IHC expression and localization of FOXO3a (Figure 6E) in hepatic sections of the control group showed few nuclear and mild, faint cytoplasmic expressions in hepatocytes; meanwhile, the DEXA group showed diffuse, high-intensity cytoplasmic expression in vacuolated and steatotic hepatocytes. On the other hand, the ENT30 group showed occasional cytoplasmic immunopositive staining in hepatocytes and the ENT60 group showed multifocal perivascular moderate intense cytoplasmic immunopositive expression in hepatocytes. The percentage of positively stained areas for FOXO3a in hepatic tissues was significantly elevated in the DEXA group compared to the control group. While co-treatment with ENT significantly reduced the percentage of positively stained areas in hepatic tissues compared to the DEXA group. Moreover, ENT60 treatment resulted in a significant decrease in hepatic expression of FOXO3a when compared to the ENT30-treated group (Figure 6F).

Furthermore, DEXA induced a significant increase in hepatic PEPCK, G6Pase and GSK with significant decrease GYS2 levels compared to control rats. ENT pre-administration (60 mg/kg) significantly decreased hepatic PEPCK, G6Pase and GSK and significantly increased GYS2 compared to the DEXA group (Figures 6G–J).

### 3.7 Effect of ENT (60 mg/kg, orally) on DEXA-induced alterations in hepatic fibrotic remodeling, EGFR signaling, and angiogenesis

TGF-β1, PAI-1, and fibronectin were assessed as markers of hepatic profibrotic signaling and extracellular matrix remodeling. EGFR was evaluated as a repair/proliferative signaling marker, while CD34 and VEGF were measured to assess endothelial remodeling and angiogenic response.

DEXA-induced a significant increase in hepatic TGF-β1, PAI-1, fibronectin, EGFR, CD34 and VEGF compared to control group. ENT (60 mg/kg, Orally) treatment significantly attenuated key pro-fibrotic signaling drivers and extracellular matrix-associated markers (TGF-β1, PAI-1, and fibronectin) compared to DEXA group. ENT treatment also was associated with a marked downregulation of pro-angiogenic pathways and vascular-related markers (VEGF, CD34, and EGFR) (Figures 7A–F).

**FIGURE 7:**
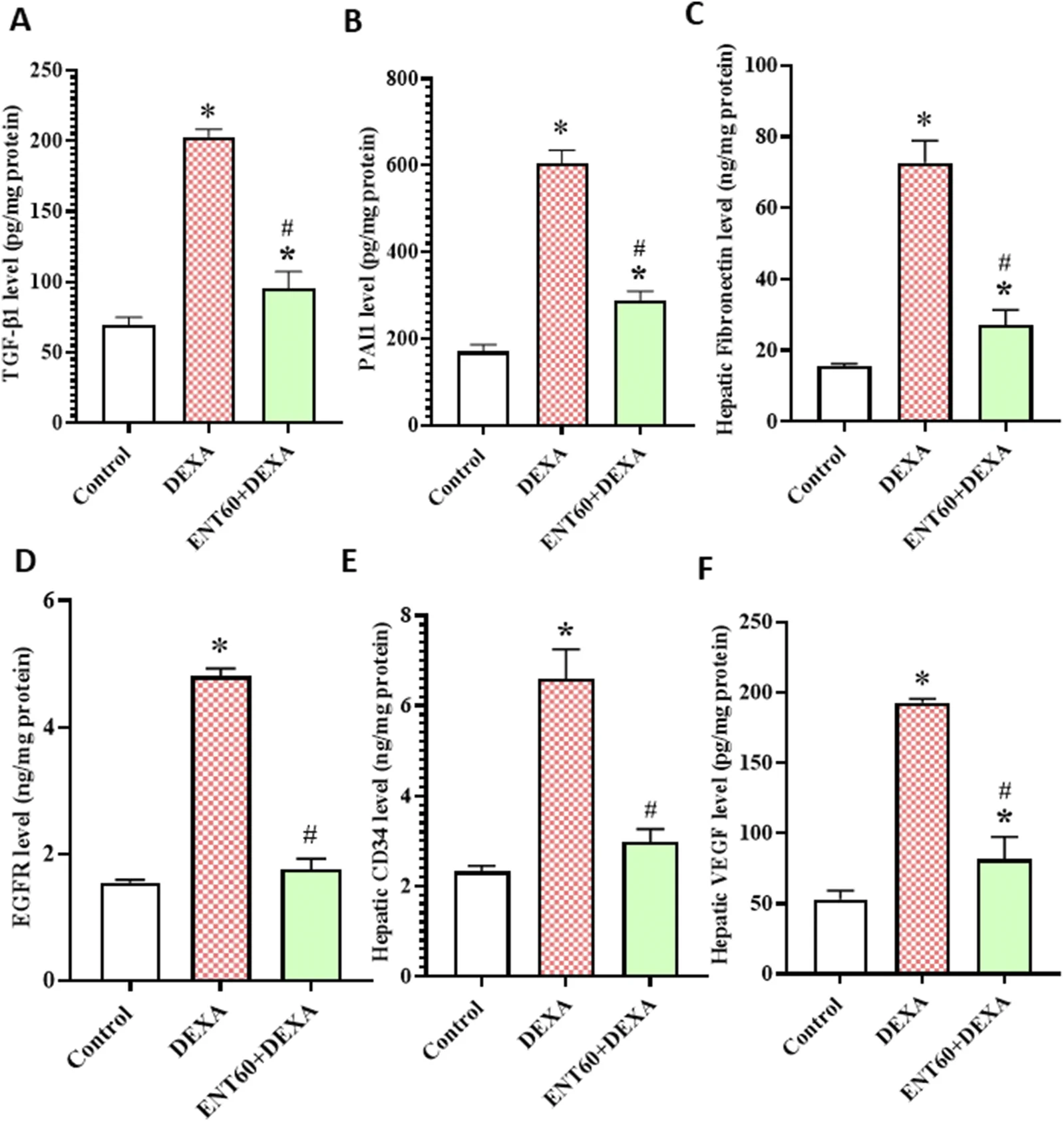
Effect of ENT (60 mg/kg, orally) on DEXA-induced alterations in hepatic fibrotic remodeling, EGFR signaling, and angiogenesis. **(A–C)** TGF-β1, PAI-1, and fibronectin were assessed as markers of profibrotic signaling and extracellular matrix remodeling. **(D)** EGFR was evaluated as a repair/proliferative signaling marker. **(E,F)** CD34 and VEGF were measured to assess endothelial remodeling and angiogenic response. Data are expressed as mean ± SD (n = 4). ENT: Entresto; DEXA: Dexamethasone; TGF-β1: Transforming growth factor-beta 1; PAI-1: Plasminogen activator inhibitor-1; EGFR: Epidermal growth factor receptor; CD34: Cluster of differentiation 34; VEGF: Vascular endothelial growth factor.

### 3.8 Effect of ENT (60 mg/kg, orally) on DEXA-induced alterations in the circulating RAAS–neprilysin–natriuretic peptide axis

DEXA administration induced a significant increase in serum Ang-II, renin, aldosterone, and circulating neprilysin protein levels compared to the control group, accompanied by a marked depletion in circulating ANP and BNP levels. Treatment with ENT (60 mg/kg, orally) in DEXA-treated rats significantly suppressed serum Ang-II, renin, aldosterone, and neprilysin protein abundance, while effectively restoring and elevating systemic ANP and BNP concentrations compared to the DEXA-alone group. These reciprocal shifts reflect a successful rebalancing of the cardiorenal-metabolic axis, favoring protective natriuretic peptide signaling over RAAS hyperactivation. Rather than establishing direct substrate cleavage kinetics, these systemic shifts reflect a favorable rebalancing of circulating natriuretic peptide availability versus RAAS activation (Figures 8A–F).

**FIGURE 8:**
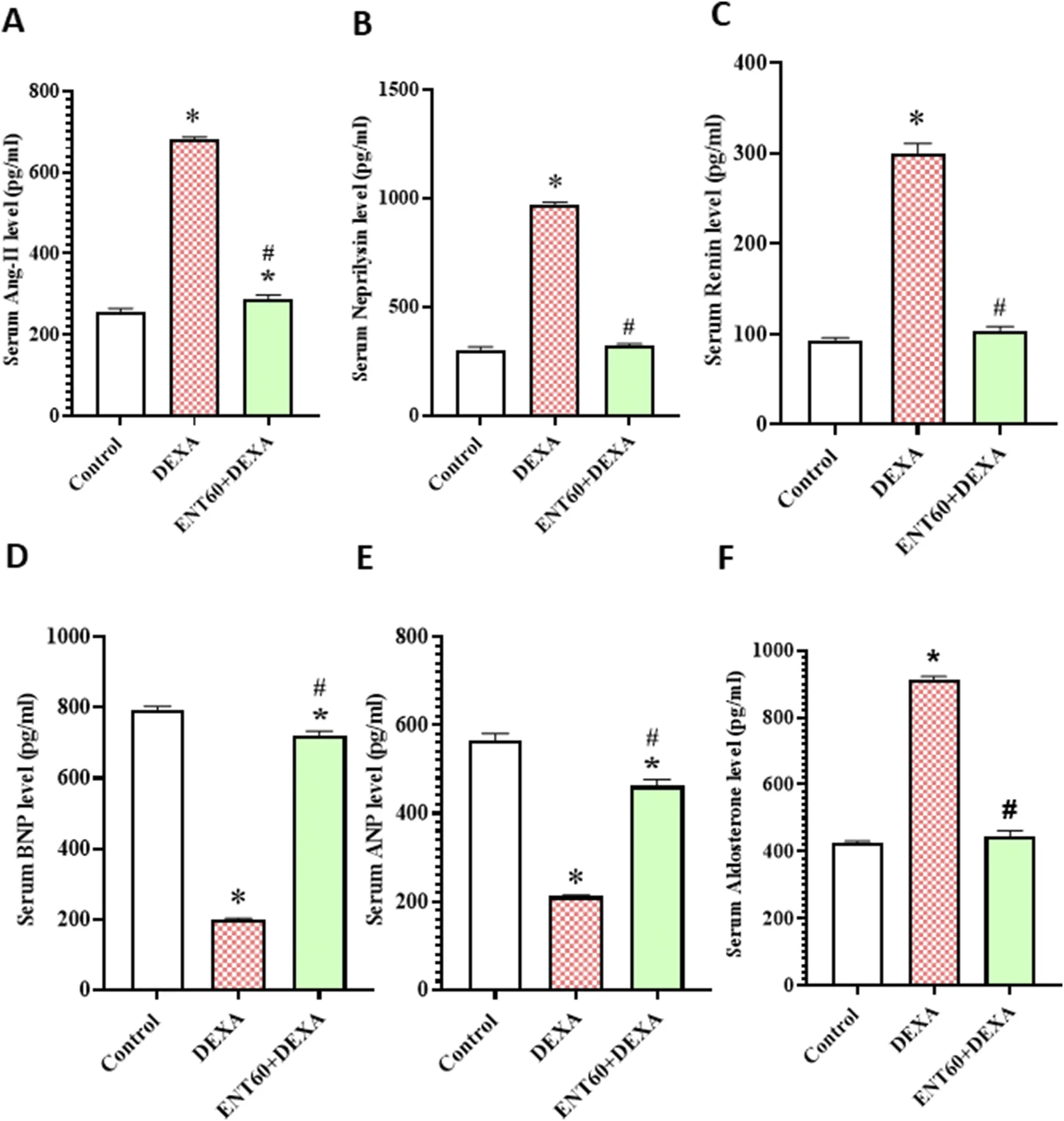
Effect of ENT (60 mg/kg, orally) on DEXA-induced alterations in the circulating RAAS–neprilysin–natriuretic peptide axis. **(A,B)** Serum renin and angiotensin II were measured to assess circulating RAAS activation. **(C)** Serum neprilysin was evaluated as a vasoactive peptide-degrading enzyme relevant to ENT pharmacology. **(D,E)** BNP and ANP were measured as circulating natriuretic peptides involved in counter-regulatory neurohormonal responses. **(F)** Aldosterone. Data are expressed as mean ± SD (n = 4). ENT: Entresto; DEXA: Dexamethasone; Ang II: angiotensin II; BNP: B-type natriuretic peptide; ANP: Atrial natriuretic peptide.

## 4 Discussion

The current study investigated the potential modulatory effects of ENT at two oral doses against DEXA-induced hepatic, metabolic and structural alterations in rats. Although glucocorticoids are well-known for their anti-inflammatory and immunosuppressive activity, sustained high dose exposure is associated with metabolic complications such as insulin resistance, hyperglycemia, lipotoxicity, and hepatic injury (Geer et at., 2014). In the current study, high-dose DEXA induced hepatic injury as evidenced by increased serum ALT, AST, and LDH activities and disrupted histopathological architecture of the liver. The current findings show glucocorticoid-induced metabolic liver injury with MASH features, and not only isolated systemic hyperglycemia.

Notably, although marked normalization of serum liver function enzymes (ALT, AST and LDH) levels and strong histopathological recovery were observed with Entresto treatment, both tested doses of ENT did not significantly change the gross liver weight or relative liver/body weight index compared to the DEXA-alone group. This observation is likely differentially related to the systemic metabolic effects of dexamethasone as opposed to the tissue protective profile of Entresto. Administration of high-dose DEXA causes marked systemic catabolism and muscle wasting, as well as considerable total weight loss, along with extreme steatotic hepatic swelling and enlargement to the point that leaves an excessive liver-to-body weight ratio (Jiao et al., 2020; Kim et al., 2022). In contrast, ENT administration maintains systemic metabolic profile (no change in calorie and protein consumption), mitigates the otherwise prevalent weight loss rates during sepsis (supporting lean body mass retention), and preserves hepatocyte health without further causing parenchymal atrophy or acute depletion of volume status. Thus, the maintenance of relative liver weight in ENT-treated groups reflects globally on systemic compensatory recovery and tissue repair, rather than impaired gastrointestinal organ toxicity *per se*, with functional biochemical and histopathological evaluations ultimately representing clear parameters in some therapeutic efficacy.

High-dose DEXA was associated with significant impairment of systemic glucose homeostasis, as evidenced by glucose intolerance, fasting hyperglycemia, hyperinsulinemia, and increased HOMA-IR. This pattern confirms the induction of glucocorticoid-induced insulin resistance. Previous studies demonstrated that IRS1/PI3K/AKT/JNK represents a pivotal role in regulating hepatic insulin sensitivity, glucose homeostasis and lipid metabolism. Dysregulation of these pathways is strongly implicated in DEXA-induced MASH (Jiang et at., 2026; Świderska et al., 2018). In the current study, DEXA reduced IRS-1 and p-AKT levels. In parallel, the increase in total JNK suggests upregulation of stress-related signaling capacity, while the reduction in p-AMPK indicates impaired hepatic energy-sensing signaling. ENT improved glucose tolerance, fasting glucose, insulin levels, and HOMA-IR, while normalizing IRS-1, p-AKT, total JNK, and p-AMPK changes. Therefore, the protective profile of ENT against DEXA-induced alteration appears to involve broad metabolic rebalancing rather than correction of a single isolated pathway.

Remarkably, acute, severe necrotic liver injury models display transient compensatory upregulation of p-AKT as an immediate cell-survival reflex (Shamsan et al., 2024), dexamethasone-induced hepatic dysfunction presents a classically different pathophysiological phenotype. Dexa acts directly on the insulin receptor signaling, inhibiting an IRS-1/PI3K/AKT axis which leads to hepatic insulin resistance, gluconeogenesis and steatosis (Beaupere et at., 2021; Chang and Wang, 2026). Thus, the marked decrease in p-AKT observed here after DEX treatment of the rats reflects severe glucocorticoid mediated insulin resistance and metabolic dysfunction. Importantly, treatment with ENT restored hepatic p-AKT phosphorylation preventing the loss of insulin signaling, reducing steatosis and mediating downstream tissue preservation.

DEXA-induced glucose intolerance was accompanied by disturbance in hepatic lipid handling and lipotoxic stress. Previous literature showed that excessive activation of gluconeogenic pathways together with enhanced *de novo* lipogenesis promotes metabolic overload within hepatocytes, leading to increased mitochondrial substrate oxidation and excessive production of ROS, thereby promoting oxidative injury and cellular dysfunction (Sunny et at., 2017). In the current study, DEXA significantly increased hepatic OxLDL, TAGs, FFA and FABP levels. This indicates enhanced fatty-acid burden, lipid oxidative modification, and altered intracellular fatty-acid transport. These changes were associated with alterations in PPAR-γ and PGC-1α, two metabolic regulators involved in lipid handling, mitochondrial adaptation and energy metabolism (Mello et at., 2016). Excessive fatty acid accumulation and oxidized lipid products are important contributors to hepatocellular stress, mitochondrial dysfunction, inflammatory signaling and progression from simple metabolic lipid accumulation toward steatohepatitis injury (Alsaedi et at., 2025; Friedman et at., 2018; Machado and Diehl, 2016). Therefore, the lipid profile observed in the DEXA group supports the development of hepatic lipotoxicity as a key component of glucocorticoid-induced metabolic liver injury.

Furthermore, hepatic TAGs accumulation in DEXA group provided direct biochemical evidence of DEXA-induced steatotic lipid deposition. This can be explained, in part, by the ability of excessive glucocorticoid exposure to promote hepatic lipid accumulation and steatotic changes. Also, DEXA-induced insulin resistance, increased peripheral lipolysis, and fatty acid influx to the liver, enhanced lipid synthesis and impaired TAGs handling or export. In line with this interpretation, previous experimental studies have shown that DEXA can promote TAGs accumulation in hepatic cells and induce hepatic steatosis *in vivo*. Therefore, the increase in hepatic TAGs observed in the current study supports the development of glucocorticoid-induced metabolic liver injury with MASH-like features (Mendoza-Figueroa et al., 1988; Kumar et al., 2015; Malkawi et al., 2018). Notably, ENT also reversed DEXA-induced lipotoxicity in the liver with significant decrease in hepatic TAGs contents. This protective effect would be explained by the potential of ENT for restoring hepatic metabolic homeostasis, possibly due to improvement in insulin sensitivity and suppression of peripheral lipolysis as well as reduction in free fatty acid influx to liver. In addition, ENT may have a role in reducing hepatic lipogenesis and stimulating hepatic lipid export pathways, which could alleviate glucocorticoid-induced steatosis. Thus, the demonstration of ENT capability restoring hepatic TAG levels reinforces its therapeutic potential on ameliorating glucocorticoid-induced metabolic liver injury and MASH-like features.

Increased lipogenesis enhances the generation of toxic lipid intermediates that stimulate lipid peroxidation and inflammatory signaling cascades, further exacerbating ROS production and hepatocellular damage (Buzzetti, Pinzani and Tsochatzis, 2016). Consistent with this concept, DEXA increased MDA and 8-OHdG while reducing SOD, indicating lipid peroxidation, oxidative DNA damage, and weakened antioxidant defense. The concomitant reduction in Nrf2 and HO-1 further suggests impairment of a key cytoprotective antioxidant pathway. These findings are in line with the recognized role of oxidative stress in the transition from metabolic hepatic stress toward inflammatory and steatohepatitis-like injury (Bellezza et at., 2018; Ma, 2013). Mitochondrial dysfunction arising from excessive fatty acid oxidation and impaired electron transport chain activity also contributes significantly to oxidative stress generation in MASLD/MASH (Walker et al., 2025).

Consistent with our findings, previous studies demonstrated that ENT attenuates oxidative stress through activation of Nrf2-dependent antioxidant signaling, suppression of ROS generation, preservation of mitochondrial integrity, and restoration of mitochondrial homeostasis (Shi et al., 2024; Zhang et al., 2024). Furthermore, ENT has been shown to enhance antioxidant defenses, reduce NF-κB-mediated oxidative injury, and improve mitochondrial quality control mechanisms in experimental disease models (Mohamad, Askar, Shaheen, Baraka and Mahmoud, 2024; Zhang et al., 2023).

In addition to oxidative stress, DEXA also significantly altered markers of mitochondrial dynamics and bioenergetic status. DEXA induced a significant increase in DRP1, which suggests mitochondrial fission or fragmentation tendency (Zhang et al., 2013). DEXA also significantly reduced ATP, which indicates impaired hepatic bioenergetic capacity. Mitochondrial dysfunction contributes to ROS generation, defective fatty acid oxidation, ATP depletion and activation of inflammatory pathways in metabolic liver disease (Begriche et al., 2013; Gusdon et al., 2014). Since p-AMPK is closely linked to cellular energy balance and mitochondrial adaptation, the concomitant disruption of p-AMPK supports disruption of hepatic energy-sensing signaling. Therefore, the combined changes in MDA, 8-OHdG, SOD, Nrf2, HO-1, DRP1, ATP and p-AMPK suggest that DEXA-induced mitochondrial dysfunction may be a result of DEXA-induced redox imbalance. Furthermore, DEXA-induced decrease in p-AMPK plays a role in triggering lipotoxicity through its close effect on PGC-1α (Chen et al., 2025).

A central, yet highly dynamic, molecular node in accelerating hepatic steatosis and progression toward MASH by DEXA is the SGK1, an early transcriptional target of the glucocorticoid receptor (GR). Its expression is deeply upregulated in hepatocytes and hepatic immune cells after DEXA administration, acting as a key mediator of downstream metabolic and fibrotic modifications (Lang et al., 2018). DEXA vigorously activates GR then binds directly to the promoter region of the *SGK1* gene, causing elevation in its transcription and enzymatic activity (Deng et al., 2009; Liu et al., 2024). High SGK1 levels change the hepatic metabolic environment. It activates the expression of lipogenic transcription factors thus enhancing *de novo* lipogenesis and the accumulation of intracellular triglycerides (Lang et al., 2018). Rising evidence highlights that DEXA-stimulated SGK1 triggers a damaging cascade in hepatic macrophages (Kupffer cells) and hepatocytes. Abundantly expressed SGK1 causing the overactivation of downstream targets as the NF-κB pathway and specific ion channels, culminating in enhanced ROS generation, mitochondrial damage, and the secretion of pro-inflammatory cytokines such as TNF-α, IL-6, and MCP-1 (Bao et al., 2022; Liu et al., 2024). This chronic inflammatory environment sustains hepatocyte ballooning and recruits systemic immune cells to the liver parenchyma. The genomic upregulation of SGK1 is strongly linked to DEXA-induced NASH. Rather than being a passive bystander, SGK1 acts as a molecular accelerator that bridges glucocorticoid signaling to lipid accumulation, oxidative stress, immune cell activation, and fibrotic remodeling (Alharbi et al., 2026). Accordingly, targeting the GR-SGK1 signaling axis offers a forceful therapeutic window to improve glucocorticoid-induced metabolic liver disorders.

Additionally, an immunohistochemical analysis showed that high-dose Entresto (60 mg/kg) significantly reduced hepatic SGK1 expression to levels just below baseline control levels. SGK1 is an essential glucocorticoid responsive kinase whose persistent activation mediates DEX-induced steatosis and metabolic dysfunction. The marked downregulation of SGK1 caused by ENT 60 demonstrates the powerful synergistic potential which dual ARNI (neprilysin inhibition and AT1 receptor antagonism) therapy has to overwhelm glucocorticoid-mediated transcriptional signals. Importantly, this potent suppression was not correlated with any negative histologic or enzymatic effect; rather it directly correlated with optimal resolution of lipid droplet content, reversal of ballooning and complete return to baseline levels for liver function markers. This suggests that the marked inhibition of SGK1 constitutes target engagement at a maximal therapeutic level along with pathway neutralization as opposed to an off-target cytotoxic occurrence.

Additionally, an immunohistochemical analysis showed that high-dose ENT (60 mg/kg) significantly reduced hepatic SGK1 expression to levels just below baseline control levels. SGK1 is an essential glucocorticoid responsive kinase whose persistent activation mediates DEXA-induced steatosis and metabolic dysfunction. The marked downregulation of SGK1 caused by ENT 60 demonstrates the powerful synergistic potential which dual ARNI (neprilysin inhibition and AT1 receptor antagonism) therapy has to overwhelm glucocorticoid-mediated transcriptional signals. Importantly, this potent suppression was not correlated with any negative histologic or enzymatic effect; rather it directly correlated with optimal resolution of lipid droplet content, reversal of ballooning and complete return to baseline levels for liver function markers. This suggests that the marked inhibition of SGK1 constitutes target engagement at a maximal therapeutic level along with pathway neutralization as opposed to an off-target cytotoxic occurrence. While our quantitative protein and immunohistochemical morphometric data demonstrate a clear, restoration of SGK1 and its downstream effectors following ENT therapy, the lack of direct genetic loss-of-function (knockdown/knockout) or gain-of-function validations precludes claiming absolute molecular causality. Consequently, the SGK1/SETD7/FOXO3a axis is best described as a promising potential regulatory pathway that coordinates with the cGMP/PKG/AMPK cascade to attenuate DEXA-induced hepatic injury.

At the metabolic output level, DEXA increased PEPCK, G6Pase and total GSK while reducing GYS2. This pattern suggests dysregulated glucose/glycogen metabolism rather than uniform activation of gluconeogenesis. The increase in PEPCK and G6Pase indicates enhanced upstream gluconeogenic drive. Reduced GYS2 with increased total GSK further suggests impaired glycogen-metabolic regulation. ENT normalized these alterations, supporting improved hepatic glucose/glycogen regulatory balance.

Macrophage activation and inflammatory polarization are increasingly recognized as important contributors to metabolic steatohepatitis-like injury and fibrotic remodeling (Wang et al., 2021). In addition to significantly increasing SETD7, an epigenetic stress mediator implicated in sustaining inflammatory gene transcription, DEXA markedly elevated TRAF1, NF-κB, and TNF-α, indicating activation of the SETD7/TRAF1/NF-κB inflammatory axis. Increased SETD7 expression may enhance chromatin accessibility and facilitate NF-κB-dependent transcription of pro-inflammatory genes, thereby amplifying cytokine production and perpetuating hepatic inflammation (Batista and Helguero, 2018; Li and Cummins, 2022). These molecular alterations were accompanied by increased CD80 and CD86, supporting activation of M1-like macrophage/APC-associated inflammatory markers, together with increased TNF-α, NF-κB, and TRAF1 immunoreactivity, indicating activation of TNF/NF-κB-related inflammatory signaling. In contrast, DEXA reduced CMAF, CD163, and CD206, suggesting suppression of macrophage-associated reparative markers. DEXA also increased CD4, IFN-γ, and hepatic IgM, suggesting involvement of adaptive and humoral immune activation. Furthermore, ENT effectively attenuated SETD7/TRAF1/NF-κB/TNF-α signaling and modulated macrophage polarization, reducing the exaggerated activation of inflammatory macrophage markers. Previous studies have shown that ENT suppresses NF-κB, JNK, and p38 MAPK signaling pathways, thereby reducing cytokine production and inflammatory tissue damage (Shi et al., 2024).

Persistent metabolic stress and inflammation were associated with activation of tissue-remodeling pathways. DEXA increased TGF-β1, PAI-1, and fibronectin, supporting activation of profibrotic and extracellular matrix-remodeling responses. In NASH, prolonged glucocorticoid insults and severe meta-inflammation trigger the hypoxia-inducible factor pathway, driving aberrant VEGF secretion to promote compensatory but dysfunctional vessel growth, while CD34 positivity marks the active recruitment and proliferation of sinusoidal endothelial cells (Ciupińska-Kajor et al., 2013; Papageorgiou et al., 2017). In the current study, DEXA-induced increase in EGFR suggests altered repair/proliferative signaling, while elevated VEGF and CD34 indicate angiogenic and endothelial remodeling responses. These findings indicate that metabolic liver injury involves crosstalk among inflammation, oxidative stress, fibrogenesis, and vascular remodeling. ENT reduced TGF-β1, PAI-1, fibronectin, EGFR, VEGF, and CD34, suggesting attenuation of profibrotic, repair-associated, and vascular remodeling responses.

Interestingly, ENT treatment significantly reduced both VEGF and CD34 levels, suggesting a potent anti-angiogenic effect. These findings are consistent with previous studies demonstrating that ENT attenuates pathological angiogenesis and vascular remodeling through suppression of RAAS activation, reduction of oxidative stress, and inhibition of pro-inflammatory signaling pathways. Moreover, ENT has been shown to decrease VEGF expression and endothelial dysfunction while improving tissue microenvironment and limiting aberrant vascular proliferation in experimental models of cardiovascular and metabolic diseases (Mohamad et al., 2024). Therefore, the reduction in VEGF and CD34 observed in the present study may represent an additional mechanism by which ENT mitigates DEXA-induced hepatic injury and prevents the progression of fibrosis and pathological tissue remodeling.

Chronic administration of DEXA induces systemic metabolic stress and volume expansion, which triggers an intrahepatic and systemic hyperactivation of the RAAS axis; this pathogenic state promotes massive aldosterone and Ang II production, directly binding to overactivated mineralocorticoid receptors in hepatic fibroblasts and immune cells to accelerate oxidative stress, inflammation, and TGF-β1-mediated fibrogenesis (Paschos and Tziomalos, 2012; Suzuki et al., 2023). In response to this DEXA-induced hypervolemia and cardiorenal-hepatic overload, the compensatory secretion of ANP and BNP is typically altered or insufficient to counteract the massive MR-driven damage (Bachmann et al., 2019; Gardner et al., 1986). Beyond secretion dysregulation, enhanced circulating protein levels or activity of neprilysin (neutral endopeptidase) a membrane-bound metalloprotease responsible for the enzymatic cleavage of active natriuretic peptides further aggravate tissue injury (Campbell, 2017; Vodovar et al., 2015). Natriuretic peptides (ANP and BNP) normally exert potent natriuretic, diuretic, vasodilatory, anti-inflammatory, and anti-fibrotic actions, alongside vital metabolic functions. Specifically, their binding to particulate guanylyl cyclase-A (GC-A) receptors increases intracellular cyclic guanosine monophosphate (cGMP) and activates downstream Protein Kinase G (PKG), promoting insulin sensitivity, AMPK catalytic activation, PGC-1α-driven mitochondrial biogenesis, fatty acid β-oxidation, and the suppression of hepatic lipogenesis and inflammation (Kuhn, 2016; Schlueter et al., 2014). Under neurohormonal stress and DEXA-induced volume overload, heightened neprilysin-mediated degradation depletes ANP/BNP bioavailability, blunting their compensatory cytoprotective and RAAS-suppressive mechanisms (Bayes-Genis, Barallat and Richards, 2016). Crucially, to verify whether restored circulating ANP and BNP levels following Entresto administration translated into functional intracellular signaling, we evaluated the downstream cGMP/PKG/AMPK cascade. In our study, Entresto treatment markedly augmented intrahepatic cGMP accumulation and downstream AMPK activation, providing direct functional validation of natriuretic peptide receptor engagement. While direct fluorometric neprilysin catalytic activity assays were not performed representing a minor study limitation the parallel restoration of circulating ANP/BNP abundance and activation of the intrahepatic cGMP/PKG/AMPK axis functionally confirm the dual action of ARNI therapy in shifting systemic and hepatic balances toward tissue-protective natriuretic signaling.

Importantly, the current findings reveal complex crosstalk between the classical Entresto-driven cardiorenal-metabolic signaling pathway and intrahepatic inflammatory machinery. Inhibition of neprilysin by sacubitril protects the degradation of natriuretic peptides (ANP/BNP) that bind to particulate guanylyl cyclase receptors and enhance intracellular cGMP accumulation and these, in turn, sequentially activated Protein Kinase G (PKG) (Kuhn, 2016). Operable PKG is an upstream physiological actor in the phosphorylation and catalytic stimulation AMPK, a master regulator of hepatic energy homeostasis. Consequentially, when activated AMPK rapidly generates a potent cross-inhibitory restraint on DEXA-induced SGK1 hyperactivation. Through inhibition of SGK1 signaling and stimulation of FOXO3a phosphorylation, the cGMP/PKG/AMPK axis ultimately prevents transcriptional engagement of pro-inflammatory programs. These upstream silencing events shut down TRAF1-dependent inflammatory cascades and block M1 pro-inflammatory macrophage recruitment, at the same time promoting a microenvironmental shift towards reparative M2 macrophages (Gong et al., 2026; Zhou et al., 2021). ENT’s therapeutic effects in MASLD extend beyond systemic hemodynamics via cGMP/PKG/AMPK orchestration, it targets intrahepatic SGK1/FOXO3a-mediated macrophage polarization from of a pro-inflammatory to an anti-inflammatory phenotype.

It is important to note that while our findings demonstrate a robust modulation of SGK1 expression, p-AKT, and FOXO3a downstream targets following ENT administration, establishing absolute molecular causality typically requires genetic loss-of-function approaches (such as SGK1 silencing or gene knockout). Previous mechanistic studies employing pharmacological SGK1 inhibitors or siRNA-mediated SGK1 knockdown have established that inhibiting SGK1 directly prevents FOXO3a hyperphosphorylation and its subsequent nuclear accumulation, thereby extinguishing gluconeogenic and pro-inflammatory gene expression in liver tissue **(** Xu et al., 2023 **)**. The parallel restoration of downstream FOXO3a transcriptional targets (G6Pase and TRAF1) and inflammatory cascades observed in our ENT-treated cohorts strongly aligns with these causal genetic models. Nevertheless, we acknowledge the lack of direct subcellular nuclear/cytosolic fractionations for FOXO3a and genetic knockdown validations as a study limitation. Ongoing follow-up studies in our laboratory using cell culture models (*in vitro* hepatocyte knockdowns) will further dissect the isolated, cell-autonomous necessity of SGK1 in mediating Entresto’s hepatoprotective actions.

In conclusion, ENT mitigates DEXA-induced metabolic liver injury with steatotic/MASH-like features in rats. This protective profile is mediated, at least in part, by restoring insulin sensitivity, normalizing hepatic triglyceride accumulation, and potential regulatory modulation of the SGK1/FOXO3a, cGMP/PKG/AMPK pathways, accompanied by a favorable shift in immune-inflammatory, pro-fibrotic, and pro-angiogenic signaling cascades. Clinical studies are needed to determine whether the hepatoprotective and metabolic effects observed experimentally can be translated into glucocorticoid-induced metabolic dysfunction and steatotic liver disease.

## 5 Limitation

Finally, several methodological limitations of the present study warrant consideration. First, while key signaling cascades (i.e., p-AKT, p-AMPK and SGK1) using a blend of ultra-sensitive quantitative ELISA and spatial immunohistochemical morphometry assays but did not perform multiplexed protein analysis and/or RT-qPCR transcriptomic profiling (to examine expression levels) at the same time points in parallel, we concede that combining these assays with Western blotting or RT-qPCR transcriptomic profiling would nevertheless enhance multi-layered validation. Fresh frozen samples were not available for immunoblotting in this part due to optimized tissue conservation formats aimed at histological preservation. Future studies will build upon these results using immunoblotting and gene-silencing approaches to dissect the sub-cellular kinetics of ARNI-mediated hepatoprotection. Second, while the parallel restoration of SGK1 immunoexpression and its functional downstream transcriptional targets (e.g., G6Pase and TRAF1) strongly aligns with established literature, direct subcellular nuclear/cytosolic fractionations for FOXO3a localization and isolated genetic loss-of-function assays (such as *in vivo*/*in vitro* SGK1 gene knockdown or knockout) were not conducted to definitively prove absolute cell-autonomous causality. Future follow-up studies will build upon these foundational results using Western blotting, transcriptomic profiling, and gene-silencing approaches to further dissect the detailed sub-cellular kinetics and functional necessity of SGK1/FOXO3a signaling in ARNI-mediated hepatoprotection.

## Data Availability

The raw data supporting the conclusions of this article will be made available by the authors, without undue reservation.
